# Integrated rehabilitation and ambulatory recovery in older adults with hip fracture according to muscle strength and sarcopenia status: a secondary analysis of a randomized controlled trial

**DOI:** 10.1007/s41999-026-01587-8

**Published:** 2026-08-20

**Authors:** Seung-Kyu Lim, Jae-Young Lim

**Affiliations:** 1 Department of Rehabilitation Medicine, Soonchunhyang University Cheonan Hospital, Soonchunhyang University College of Medicine, Cheonan, Republic of Korea; 2 Department of Rehabilitation Medicine, Seoul National University Bundang Hospital, Seoul National University College of Medicine, 82 Gumi-Ro 173Beon-Gil, Bundang-Gu, Seongnam, Gyeonggi-Do 13620 Republic of Korea; 3 Institute on Aging, Seoul National University, Seoul, Republic of Korea

**Keywords:** Hip fracture, Older people, Sarcopenia, Rehabilitation, Muscle strength

## Abstract

**Purpose:**

Sarcopenia and impaired muscle strength are common in older adults with hip fractures and are strongly associated with poor functional recovery. Although integrated multidisciplinary rehabilitation improves outcomes after hip fracture, whether its benefits differ according to baseline muscle strength or sarcopenia severity remains unclear. This study aimed to examine whether the functional benefits of Fragility Fracture Integrated Rehabilitation Management (FIRM) vary according to sarcopenia status and handgrip strength (HGS) in older adults after hip fracture surgery.

**Methods:**

This was a secondary analysis of a multicenter randomized controlled trial comparing FIRM with conventional rehabilitation in adults aged ≥ 65 years undergoing hip fracture surgery. The primary outcome was independent ambulation at 12 months. Sarcopenia was classified using three approaches: sarcopenia versus non-sarcopenia, a three-level classification (non-sarcopenia, possible sarcopenia, and sarcopenia), and low versus normal HGS. Logistic regression models, including treatment, sarcopenia status, and their interactions, were fitted with sequential adjustments for relevant covariates.

**Results:**

Of the 203 randomized participants, 119 (58.6%) completed the 12-month follow-up. Treatment-by-sarcopenia interactions were not statistically significant. Stratum-specific analyses suggested a pattern of greater functional improvement with FIRM than with conventional rehabilitation, particularly among participants with low HGS and, to a lesser extent, those with sarcopenia. In the fully adjusted models, FIRM was associated with greater improvement in independent ambulation among participants with low HGS (odds ratio, 4.25; risk difference, + 34.7%; *P* = 0.014).

**Conclusions:**

Integrated rehabilitation may provide greater functional benefits than conventional rehabilitation in older adults with hip fractures, particularly among those with low muscle strength. However, given the lack of statistically significant interaction effects and the attenuation of the observed associations in the fully adjusted models, these findings should be interpreted as exploratory.

**Supplementary Information:**

The online version contains supplementary material available at https://doi.org/10.1007/s41999-026-01587-8.

## Key summary points

**Aim:**

To examine whether the functional benefits of integrated rehabilitation vary by sarcopenia status and handgrip strength in older adults with hip fracture

**Findings:**

Functional recovery differed according to baseline muscle strength and sarcopenia status. Older adults with sarcopenia or low handgrip strength appeared to show greater ambulatory improvement with integrated rehabilitation than with conventional rehabilitation.

**Message:**

Low muscle strength may identify a vulnerable yet rehabilitation-responsive subgroup, and early assessment may help guide stratified rehabilitation after hip fracture.

**Supplementary Information:**

The online version contains supplementary material available at https://doi.org/10.1007/s41999-026-01587-8.

## Introduction

Hip fractures in older adults are associated with a poor prognosis and represent a substantial clinical and socioeconomic burden [1], particularly in rapidly aging societies such as Korea [2]. As the population ages, multiple age-related factors contribute to the occurrence of fragility fractures [3], among which sarcopenia plays a critical role in the development of hip fractures and in their subsequent recovery [3, 4].

Sarcopenia, a progressive and generalized skeletal muscle disorder characterized by the loss of muscle mass and function, is common in older adults and strongly associated with adverse clinical outcomes [5]. Its prevalence is estimated to range between 5 and 10% in the general population and between 10 and 22% in older adults [5, 6], with higher rates reported in Asian populations (13–19%) [7], particularly in Korea, where nearly 23% of adults aged ≥ 70 years are affected [8]. Sarcopenia is more prevalent among institutionalized individuals and those with specific clinical conditions, including cancer and critical illness [5, 6]. Among patients with hip fractures, sarcopenia is markedly more common than in those with other fragility fractures, with a reported prevalence of 12‒95% among males and 18‒64% among females [9]. Importantly, sarcopenia is a key adverse prognostic factor in this population and is associated with increased short- and long-term mortality, higher rates of postoperative complications and infections, prolonged hospitalization, and poorer functional recovery [10–12], substantially complicating postoperative rehabilitation [13].

Integrated rehabilitation strategies, particularly resistance exercises combined with nutritional interventions, have been proposed as key approaches for improving outcomes in older adults with sarcopenia [5, 14, 15]. In patients with hip fractures and sarcopenia, recovery likely requires a comprehensive approach that addresses both muscle mass preservation and functional improvement [16, 17]. Previous studies have shown that moderate- to high-intensity resistance exercise and structured intensive rehabilitation can improve function and walking ability after hip fractures in older adults, irrespective of sarcopenia status [18, 19]. Nevertheless, whether intensive multidisciplinary rehabilitation programs confer additional benefits over conventional rehabilitation, particularly in patients with sarcopenia, remains unclear.

Fragility Fracture Integrated Rehabilitation Management (FIRM) is a multidisciplinary integrated rehabilitation program that has demonstrated superior functional outcomes compared with conventional rehabilitation in older adults with hip fractures over a 12-month postoperative follow-up period in a multicenter randomized controlled trial (RCT) [20]. However, the potential heterogeneity in treatment response, particularly among frail or vulnerable patients such as those with impaired muscle strength or varying severity of sarcopenia, has not been fully explored. Therefore, in this secondary analysis of an RCT, we aimed to examine whether the effectiveness of FIRM in improving 12-month ambulatory recovery differs according to baseline sarcopenia status and muscle strength in older adults undergoing hip fracture surgery, compared with conventional rehabilitation.

## Methods

### Study design and participants

This study was a secondary analysis of a multicenter, parallel-group, single-blind RCT comparing FIRM with conventional rehabilitation in older adults after hip fracture surgery, with a 12-month follow-up [20]. The trial was conducted between February 2017 and February 2020. The study protocol was approved by the Institutional Review Boards of Seoul National University Bundang Hospital (B-1603–337-002), Chung-Ang University Hospital (C2016117[1860]), and Jeju National University Hospital (JEJUNUH 2016–11-001). All participants provided written informed consent prior to enrollment.

Participants aged ≥ 65 years who underwent surgical treatment for hip fractures were randomized to receive either FIRM or conventional rehabilitation. The detailed eligibility criteria and randomization procedures have been previously reported [20]. All randomized participants were included in the secondary analysis. Participants without baseline handgrip strength (HGS) and muscle mass measurements could not be classified according to sarcopenia status and were therefore excluded from the sarcopenia-stratified analyses, but were included in the overall cohort description.

### Data collection

Baseline sociodemographic and clinical data were collected using standardized questionnaires and medical record reviews. Variables included demographic characteristics, fracture- and surgery-related factors, comorbidities assessed using the Charlson Comorbidity Index (CCI) [21], pre-fracture walking ability, length of hospital stay, time to surgery, time to transfer to the rehabilitation unit, and discharge destination. Walking ability was assessed using the Koval scale [22] and the Functional Ambulation Category (FAC) [23]. Pre-fracture independent ambulation was defined as the ability to walk outdoors without physical assistance, regardless of assistive device use (Koval score ≤ 3 and FAC score ≥ 3); failure to meet this criterion was considered pre-fracture gait impairment. Cognitive impairment was evaluated using the Mini-Mental State Examination (MMSE) [24], with cutoffs adjusted for age, sex, and education level [25].

### Sarcopenia classification

Muscle mass was assessed using bioelectrical impedance analysis (BIA). Appendicular skeletal muscle mass (ASM) was calculated using the lean mass from both the upper and non-fractured lower extremities to minimize bias related to postoperative edema or orthopedic implants [26]. The ASM index (ASMI) was calculated as ASM divided by height squared (kg/m^2^). BIA measurements were performed after transfer to the rehabilitation unit using a tetrapolar system (InBody S10; Biospace, Seoul, Korea), according to standardized protocols [27]. HGS was measured using a digital dynamometer (TKK 5401 Grip-D; Takei, Niigata, Japan), according to established procedures [28].

Sarcopenia status was retrospectively classified according to the 2025 consensus of the Asian Working Group for Sarcopenia (AWGS), using available baseline data. Baseline sarcopenia was defined based on the criteria for low muscle strength (HGS < 28 kg in males and < 18 kg in females) and low muscle mass (ASMI < 7.0 kg/m^2^ in males and < 5.7 kg/m^2^ in females) [14]. Muscle mass measurements were unavailable for some participants, primarily owing to the limited feasibility of BIA during the early stages of rehabilitation at certain participating centers. Two classification approaches were applied. First, among participants with complete data, sarcopenia was defined as the concurrent presence of low HGS and low muscle mass. Second, to retain participants with available HGS data despite missing muscle mass measurements, a three-level classification was used: non-sarcopenia (normal HGS), possible sarcopenia (low HGS without muscle mass measurement), and sarcopenia (concurrent low HGS and low muscle mass). Participants with low HGS but missing muscle mass measurements were classified as having possible sarcopenia. These subgroup analyses were not prespecified and were conducted as part of a secondary exploratory analysis.

### Intervention

Participants in the intervention group underwent FIRM, a structured, multidisciplinary rehabilitation program combining physiotherapy (PT), occupational therapy (OT), and patient and caregiver education linked to community-based rehabilitation services. The FIRM program consisted of two 30-min PT sessions per day on weekdays over a 2-week inpatient rehabilitation period, along with four OT sessions during admission. PT focused on progressive weight-bearing and functional mobility training, incorporating individualized strengthening, balance, gait, and aerobic exercises with gradual progression based on patient tolerance and functional status. OT targeted activities of daily living (ADLs), including transfers, bed mobility, dressing, self-care, and use of assistive devices. In addition, structured education was provided to both patients and caregivers, covering exercise progression, fall prevention, safety, and discharge planning. A dietitian performed the nutritional assessment and delivered individualized counseling, including recommendations for adequate caloric and protein intake and dietary strategies during hospitalization and after discharge. However, the program did not include standardized nutritional supplementation or protocolized nutritional interventions.

Participants in the conventional rehabilitation group received a less intensive program consisting of a single 30-min PT session per day focused on upright tolerance and basic gait training, along with standard fall-prevention counseling and routine postoperative care. All participants completed a 2-week inpatient rehabilitation program. The detailed intervention protocols have been reported previously [20].

### Outcome

Independent ambulation at 12 months was defined as a FAC score ≥ 3 combined with a Koval walking ability score ≤ 3, consistent with the pre-fracture ambulation criteria.

### Statistical analysis

Baseline characteristics of the randomized cohort were summarized by treatment group using means with standard deviations for continuous variables and counts with percentages for categorical variables. The comparative effectiveness of FIRM versus conventional rehabilitation across sarcopenia strata was evaluated using logistic regression, with independent ambulation at 12 months as the outcome. The models included the treatment group, sarcopenia status, and their interaction; treatment effects were reported as stratum-specific odds ratios (ORs) with 95% confidence intervals (CIs). Three analytical models were applied: Model 1 included participants with complete baseline HGS and muscle mass data (sarcopenia vs. non-sarcopenia) and served as a complete-case analysis to assess the robustness of the findings; Model 2 applied a three-level AWGS-based classification among participants with available HGS (non-sarcopenia, possible sarcopenia, and sarcopenia); and Model 3 dichotomized participants by muscle strength alone (normal vs. low HGS), regardless of muscle mass availability.

Stratum-specific ORs, model-based predicted probabilities, and absolute risk differences (RDs) were estimated using marginal means, and the number needed to treat (NNT) was calculated as the inverse of the RD. Analyses were performed sequentially using unadjusted, age- and sex-adjusted, and fully adjusted models. The fully adjusted models additionally included cognitive impairment, CCI, pre-fracture gait impairment, and variables showing baseline imbalance. To assess the potential impact of attrition bias, baseline characteristics were compared between participants with and without a 12-month follow-up. In addition, sensitivity analyses were performed for Model 3 among all participants with available baseline HGS data, using both worst-case and best-case assumptions for missing 12-month outcomes. In the worst-case scenario, all participants lost to follow-up were assumed to be non-ambulatory, whereas in the best-case scenario, they were assumed to be ambulatory. All tests were two-sided, with a significance level of 0.05. Analyses were conducted using R software (version 4.4.3; R Foundation for Statistical Computing, Vienna, Austria).

## Results

### Characteristics of the participants

**Fig. 1:**
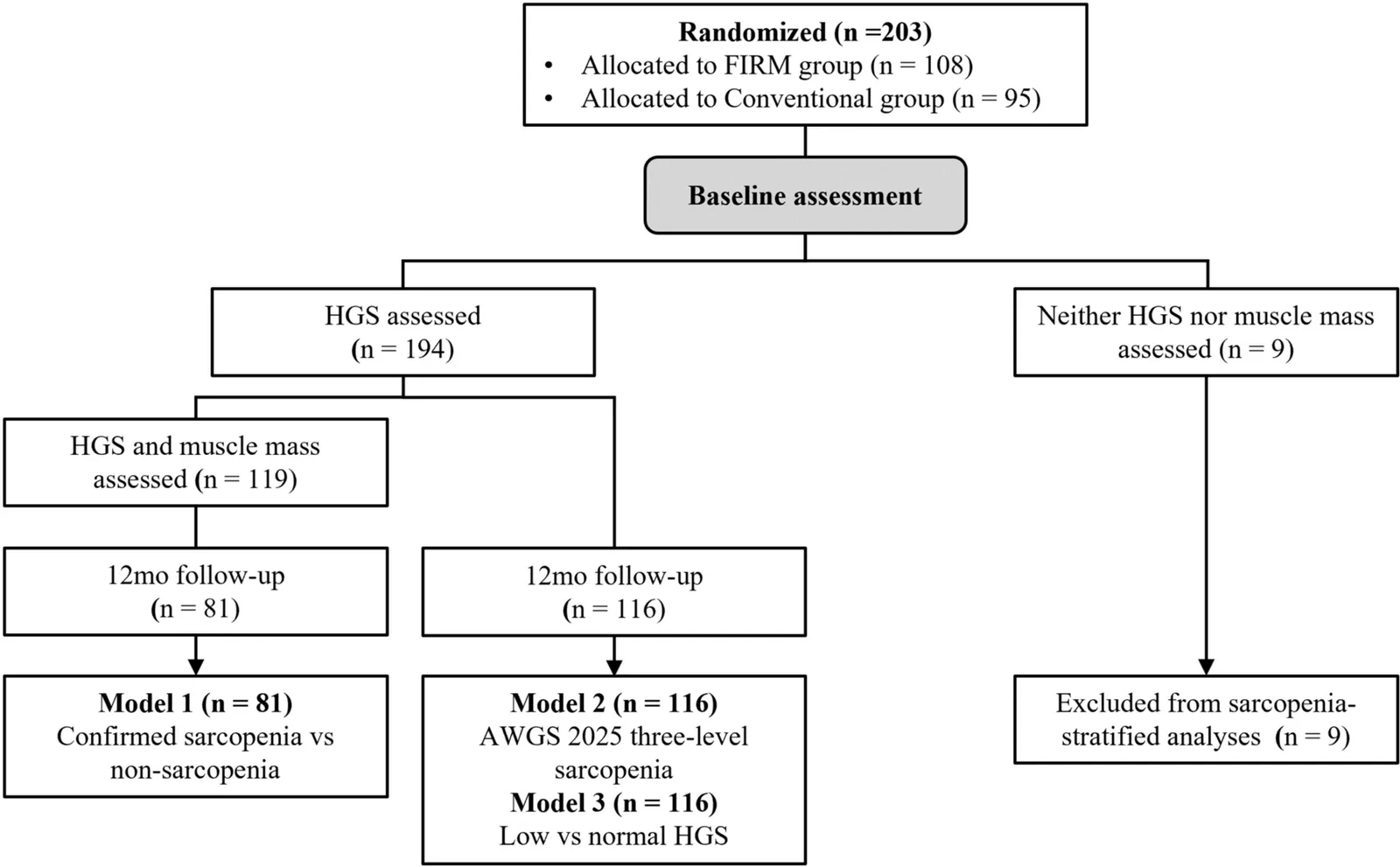
Flow of participants and availability of sarcopenia-related measurements. A total of 203 participants were randomized. At baseline, HGS was available for 194 participants, of whom 119 also had muscle mass measurements. Among participants with 12-month outcome data, baseline HGS data were available for 116 participants, who were included in Models 2 and 3; both baseline HGS and muscle mass data were available for 81 participants, who were included in Model 1. FIRM, Fragility Fracture Integrated Rehabilitation Management; HGS, handgrip strength

The clinical characteristics of the randomized cohort are summarized in Table 1, and the participant flow, including eligibility for sarcopenia-stratified analyses, is shown in Fig. 1. A total of 203 patients were randomized to the FIRM (n = 108) or conventional rehabilitation (n = 95) groups, and 119 (58.6%) completed the 12-month follow-up. Detailed reasons for dropout and loss to follow-up were previously reported in the original randomized controlled trial publication [20]. As shown in Fig. 1, nine participants lacked baseline HGS and muscle mass data, and muscle mass data were missing for an additional 75 participants. The baseline characteristics were generally comparable between the groups, except for a longer time to transfer to the rehabilitation unit and a lower prevalence of cognitive impairment in the FIRM group. Among the 119 participants with 12-month follow-up, the proportion of independent ambulation was higher in the FIRM group than in the conventional rehabilitation group (53/69 [76.8%] vs. 28/50 [56.0%], *P* = 0.027).

**Table 1:** Baseline characteristics of the randomized cohort according to treatment group

Variable	FIRM group (n = 108)	Conventional group (n = 95)	*P-*value
Age, mean (SD), years	80.8 (7.3)	82.0 (6.0)	0.212
Sex, n (%) (female)	82 (75.9)	73 (76.8)	0.878
Height, mean (SD), cm	155.5 (8.5)	156.0 (8.5)	0.733
Weight, mean (SD), kg	53.3 (9.4)	55.1 (10.0)	0.188
BMI, mean (SD), kg/m^2^	22.1 (3.7)	22.6 (3.4)	0.276
ASMI, mean (SD), kg/m^2^ (n = 119)			
Male	6.3 (1.2)	6.2 (1.2)	0.778
Female	5.3 (1.0)	5.7 (1.0)	0.120
HGS, mean (SD), kg (n = 194)			
Male	20.6 (8.6)	22.0 (7.2)	0.561
Female	13.9 (6.0)	13.6 (6.3)	0.803
Fracture type, n (%)			0.103
Femoral neck	50 (46.3)	32 (33.7)	
Intertrochanteric	51 (47.2)	59 (62.1)	
Subtrochanteric	7 (6.5)	4 (4.2)	
Operation type, n (%)			0.014
Bipolar hemiarthroplasty	47 (43.5)	52 (54.7)	
Total hip arthroplasty	11 (10.2)	1 (1.1)	
Internal fixation	50 (46.3)	42 (44.2)	
Pre-fracture Koval, mean (SD), score	1.7 (1.2)	1.8 (1.1)	0.794
Pre-fracture FAC, mean (SD), score	4.6 (0.8)	4.6 (0.6)	0.562
Pre-fracture gait impairment, n (%)	12 (11.1)	8 (8.4)	0.521
Previous history of hip fracture, n (%)	7 (6.5)	5 (5.3)	0.713
Cognitive impairment, n (%)	39 (36.1)	47 (49.5)	0.046
Osteoporosis, n (%)	77 (71.3)	70 (73.7)	0.704
Charlson Comorbidity Index, n (%)			0.913
None	37 (34.3)	33 (34.7)	
Mild (1–2)	51 (47.2)	41 (43.2)	
Moderate (3–4)	9 (8.3)	9 (9.5)	
Severe (≥ 5)	11 (10.2)	12 (12.6)	
Postoperative complication, n (%)	3 (2.8)	8 (8.4)	0.076
Length of hospital stay, mean (SD), days	24.0 (6.4)	23.4 (6.3)	0.537
Days to surgery, mean (SD), days	2.9 (2.3)	3.2 (3.7)	0.438
Time to transfer to the rehabilitation unit, mean (SD), days	8.4 (3.7)	7.0 (2.7)	0.003
Discharge location, n (%)			0.969
Home	56 (51.9)	49 (51.6)	
Institution	52 (48.1)	46 (48.4)	

*FIRM* Fragility Fracture Integrated Rehabilitation Management; *BMI* body mass index; *ASMI* appendicular skeletal muscle mass index; *HGS* handgrip strength; *FAC* functional ambulation category

The clinical characteristics stratified by sarcopenia status among participants with available HGS data (n = 194) are presented in Supplementary Table S1. The distribution of the rehabilitation groups did not differ significantly across sarcopenia categories. Participants with possible sarcopenia or sarcopenia were older and had a lower body mass index, poorer baseline functional status, a higher comorbidity burden, and a higher prevalence of pre-fracture gait impairment, prior hip fracture, cognitive impairment, and institutional discharge, indicating clinically meaningful differences across the sarcopenia spectrum. Among the 116 participants with available baseline HGS data, the rate of independent ambulation at 12 months decreased across the sarcopenia categories (non-sarcopenia, 31/36 [86.1%]; possible sarcopenia, 20/31 [64.5%]; sarcopenia, 28/49 [57.1%]; *P* = 0.016).

### Comparative effectiveness of FIRM versus conventional rehabilitation across the sarcopenia strata

#### Model 1: Sarcopenia vs non-sarcopenia

Among the 81 participants with complete sarcopenia classification, 49 had sarcopenia and 32 did not. In the sarcopenia group, independent ambulation at 12 months was achieved in 21 of 31 (67.7%) participants in the FIRM group and 7 of 18 (38.9%) participants in the conventional group. In the non-sarcopenia group, independent ambulation was achieved in 14 of 16 (87.5%) participants in the FIRM group and 13 of 16 (81.3%) participants in the conventional group.

**Fig. 2:**
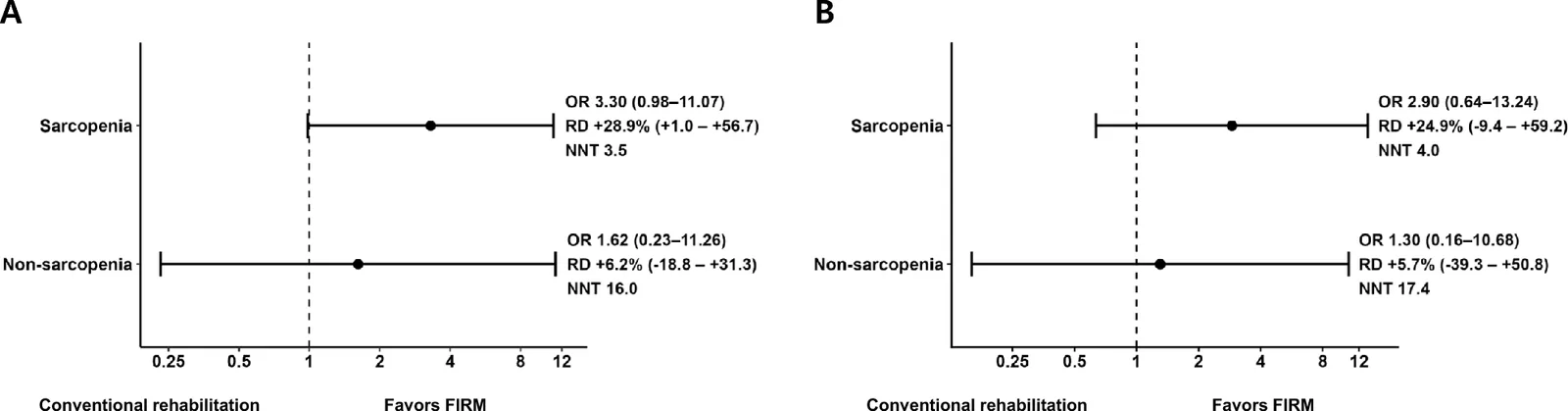
Effect of FIRM on 12-month independent ambulation by sarcopenia status. Forest plots show the effect of FIRM compared with conventional rehabilitation on independent ambulation at 12 months, stratified by sarcopenia status. Panel A shows unadjusted estimates, and panel B shows fully adjusted estimates controlling for age, sex, cognitive impairment, the Charlson Comorbidity Index, and pre-fracture gait impairment. Treatment effects are presented as odds ratios (ORs), risk differences (RDs), and numbers needed to treat (NNTs). FIRM, Fragility Fracture Integrated Rehabilitation Management

The treatment-by-sarcopenia interaction was not statistically significant in the unadjusted or adjusted models (all *P* for interaction > 0.54). However, stratum-specific analyses suggested that the benefits of FIRM may differ according to sarcopenia status (Fig. 2). In the unadjusted analyses, FIRM was associated with a greater absolute increase in independent ambulation at 12 months in the sarcopenia group (OR, 3.30; RD, + 28.9%; *P* = 0.043; NNT = 3.5), whereas no significant difference was observed in the non-sarcopenia group (*P* = 0.625). This pattern persisted after adjusting for age and sex (OR, 3.39; RD, + 29.1%; *P* = 0.042; NNT = 3.4) but was attenuated and no longer statistically significant in the fully adjusted model (*P* = 0.155) (Fig. 2 and Supplementary Fig. S2).

#### Model 2: Three-level AWGS 2025 sarcopenia classification

Among the 116 participants with available baseline HGS data, the crude rate of independent ambulation at 12 months declined across the sarcopenia categories. In the non-sarcopenia group, independent ambulation was achieved in 17 of 19 (89.5%) participants in the FIRM group and 14 of 17 (82.4%) participants in the conventional group. In the possible sarcopenia group, 13 of 16 (81.3%) participants in the FIRM group and 7 of 15 (46.7%) participants in the conventional group achieved independent ambulation. In the sarcopenia group, independent ambulation was observed in 21 of 31 (67.7%) participants in the FIRM group and 7 of 18 (38.9%) participants in the conventional group.

**Fig. 3:**
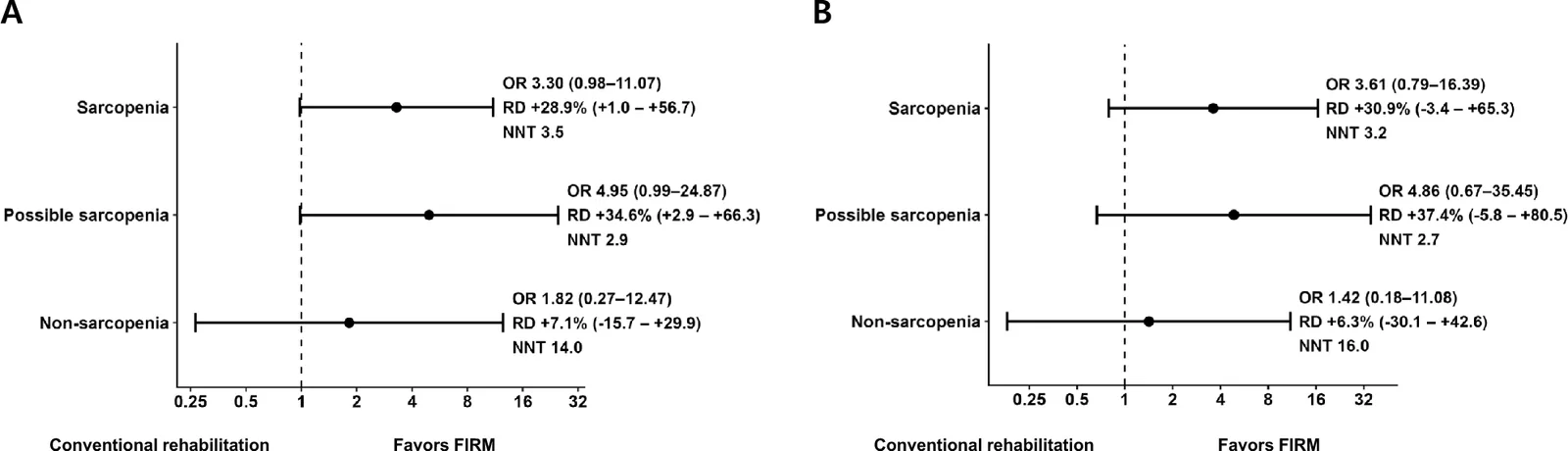
Effect of FIRM on 12-month independent ambulation across the AWGS 2025 sarcopenia classification. Forest plots show stratum-specific treatment effects of FIRM compared with conventional rehabilitation on independent ambulation at 12 months across three sarcopenia categories (non-sarcopenia, possible sarcopenia, and sarcopenia). Panel A shows unadjusted estimates, and panel B shows fully adjusted estimates controlling for age, sex, cognitive impairment, the Charlson Comorbidity Index, and pre-fracture gait impairment. Treatment effects are presented as odds ratios (ORs), risk differences (RDs), and numbers needed to treat (NNTs). FIRM, Fragility Fracture Integrated Rehabilitation Management

Although the treatment-by-sarcopenia interaction was not statistically significant (all *P* > 0.67), stratum-specific analyses suggested that the effect of FIRM may differ across sarcopenia categories (Fig. 3). In the unadjusted analyses, FIRM was associated with greater absolute improvements in independent ambulation among participants with possible sarcopenia (OR, 4.95; RD, + 34.6%; *P* = 0.032; NNT = 2.9) and sarcopenia (OR, 3.30; RD, + 28.9%; *P* = 0.043; NNT = 3.5), but not in those without sarcopenia (*P* = 0.54). After adjusting for age and sex, FIRM remained associated with greater independent ambulation among participants with sarcopenia (OR, 3.78; RD, + 30.9%; *P* = 0.030; NNT = 3.2), whereas the estimated effect was attenuated among those with possible sarcopenia. In the fully adjusted model, the estimated effects remained numerically greater in the possible sarcopenia (OR, 4.86; RD, + 37.4%; *P* = 0.090; NNT = 2.7) and sarcopenia groups (OR, 3.61; RD, + 30.9%; P = 0.078; NNT = 3.2). However, no statistically significant differences were observed, and no significant difference was observed in the non-sarcopenia group (*P* = 0.736) (Fig. 3 and Supplementary Fig. S2).

#### Model 3: Low versus normal HGS

**Fig. 4:**
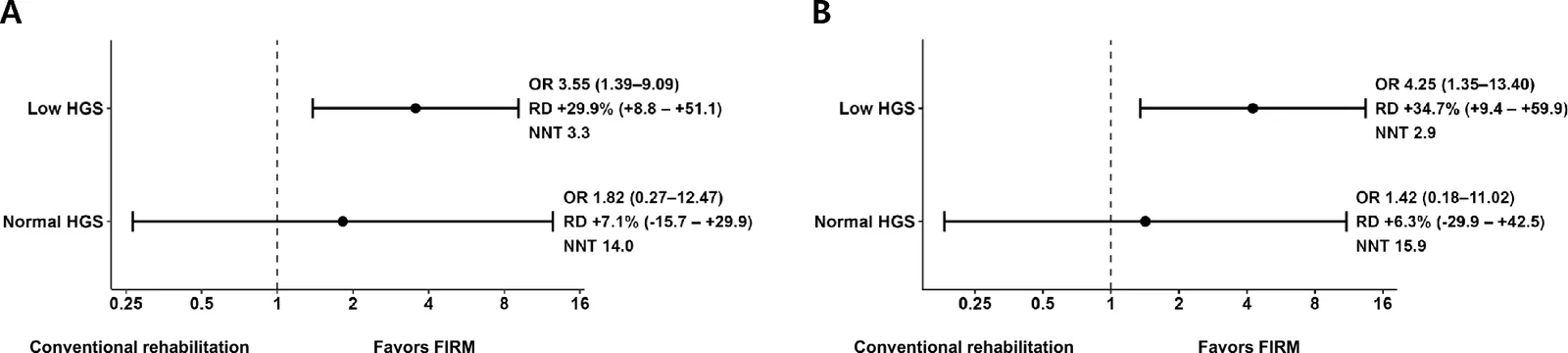
Effect of FIRM on 12-month independent ambulation by handgrip strength status. Forest plots show the effect of FIRM compared with conventional rehabilitation on independent ambulation at 12 months, stratified by baseline handgrip strength status (normal vs. low). Panel A shows unadjusted estimates, and panel B shows fully adjusted estimates controlling for age, sex, cognitive impairment, the Charlson Comorbidity Index, and pre-fracture gait impairment. Treatment effects are presented as stratum-specific odds ratios (ORs), risk differences (RDs), and numbers needed to treat (NNTs). HGS, handgrip strength; FIRM, Fragility Fracture Integrated Rehabilitation Management

Among the 116 participants with available baseline HGS data, the distribution of independent ambulation at 12 months in the normal HGS group was similar to that observed in the non-sarcopenia group in Model 2. In the low HGS group, independent ambulation was achieved in 34 of 47 (72.3%) participants in the FIRM group and 14 of 33 (42.4%) participants in the conventional group. When participants were dichotomized by baseline HGS, the treatment-by-strength interaction was not statistically significant (*P* for interaction = 0.41–0.55). However, the stratum-specific analyses suggested that the benefits of FIRM may differ according to baseline muscle strength (Fig. 4).

In the unadjusted analyses, FIRM was associated with a greater absolute increase in independent ambulation at 12 months among participants with low HGS (OR, 3.55; RD, + 29.9%; *P* = 0.006; NNT = 3.3), with no meaningful benefit observed in those with normal strength (*P* = 0.54). This finding persisted even after adjusting for age and sex (OR, 3.65; RD, + 28.9%; *P* = 0.007; NNT = 3.5) and remained consistent in the fully adjusted model (OR, 4.25; RD, + 34.7%; *P* = 0.014; NNT = 2.9), whereas no significant differences were observed in the normal HGS group (*P* = 0.737) (Fig. 4 and Supplementary Fig. S2).

In all fully adjusted models, several baseline variables, including cognitive impairment, comorbidity burden, and pre-fracture functional status, were independently associated with the 12-month functional outcomes, whereas operation type and time to rehabilitation transfer were not. Additional adjustments for these baseline imbalances attenuated some of the estimated treatment effects across the models, although the overall patterns remained consistent.

Baseline characteristics of participants with and without 12-month follow-up are presented in Supplementary Table S3. Participants who were lost to follow-up were older, had lower body weight, BMI, and HGS, and were more likely to be discharged to institutional care, suggesting a more vulnerable clinical profile and a potentially lower likelihood of functional recovery. Sensitivity analyses using worst-case assumptions showed attenuation of the estimated effects, although the overall pattern remained consistent, with a greater benefit from FIRM among participants with low HGS. Similar directional findings were observed in the best-case analyses, although the effect sizes were reduced and did not consistently reach statistical significance (Supplementary Figs. S4 and S5).

## Discussion

In this secondary analysis of a multicenter RCT, we found that the functional benefit of integrated rehabilitation after hip fracture was not uniform but varied according to baseline muscle strength and, to a lesser extent, sarcopenia status, although these patterns should be interpreted cautiously. Although formal tests of treatment-by-sarcopenia interactions were not statistically significant, stratum-specific analyses suggested differences in estimated treatment effects across analytical models. In particular, older adults with low HGS appeared to derive greater ambulatory benefits from FIRM than from conventional rehabilitation, as reflected by greater absolute gains in independent ambulation and a relatively low NNT at 12 months. In contrast, although similar patterns were observed among participants with sarcopenia, the estimated effects were attenuated and did not consistently reach statistical significance in the fully adjusted models. These findings should be interpreted in the context of additional adjustments for baseline imbalances, which attenuated some of the estimated effects. Importantly, because the interaction terms were not statistically significant, these findings should be considered exploratory and hypothesis-generating rather than confirmatory.

Sarcopenia is a critical condition in older adults with hip fractures and serves as both a contributor to fracture occurrence and a major barrier to postoperative recovery. It reflects a complex multifactorial pathophysiology, including reduced mechanical loading, impaired muscle protein synthesis, chronic inflammation, and metabolic dysregulation, resulting in a concurrent decline in muscle strength and bone quality [3, 29]. Consequently, sarcopenia impairs gait and balance, increases the risk of falls and fractures [3, 29, 30], and is consistently associated with delayed functional recovery and poor prognosis after hip fracture [10–13]. Importantly, sarcopenia is at least partially reversible [31], underscoring the clinical relevance of early identification and timely implementation of targeted interventions. Given its dual role as both a precipitating factor and a modifiable determinant of recovery, sarcopenia should be considered a central therapeutic target in post-hip fracture care.

Our findings suggest that baseline muscle strength and sarcopenia status may be critical determinants of appropriate rehabilitation strategies for vulnerable patients after hip fracture surgery. Multidisciplinary, intensive, and structured rehabilitation has been shown to improve functional outcomes, reduce institutionalization, and decrease mortality in this population [32, 33]. However, despite sarcopenia being a well-established poor prognostic factor, comparative evidence for optimal rehabilitation strategies in this population remains limited. Previous studies have largely focused on individual exercises or nutritional components as adjuncts to conventional rehabilitation, with heterogeneous designs and a limited comparative scope [34–37]. In this context, the present study provides comparative evidence that an integrated multidisciplinary rehabilitation program may confer greater functional benefits than conventional rehabilitation in older adults with hip fractures, sarcopenia, or impaired muscle strength. Across multiple analytical models, this advantage was reflected in greater absolute gains in independent ambulation, with the benefit of integrated rehabilitation appearing more evident in participants with low muscle strength and, to a lesser extent, in those with sarcopenia. Notably, despite substantial functional vulnerability, these patients appeared to retain their rehabilitation potential when exposed to intensive integrated interventions. However, the use of multiple classification approaches, including a possible sarcopenia category based on HGS alone, reflects pragmatic constraints in postoperative settings and introduces uncertainty in classification.

These findings also suggest that conventional rehabilitation alone may be insufficient to achieve optimal functional recovery in patients with sarcopenia or impaired muscle strength, as it may not adequately address the complex physical and functional deficits inherent to sarcopenia. These findings should not be interpreted as evidence that integrated rehabilitation has no benefit in patients with preserved muscle strength. Instead, our results suggest that integrated multidisciplinary rehabilitation may be particularly beneficial for maximizing recovery in patients with sarcopenia or low muscle strength. From a clinical perspective, these data support consideration of a stratified rehabilitation approach in which patients with impaired muscle strength are proactively identified and prioritized for intensive programs such as FIRM to optimize ambulatory recovery after hip fracture.

Recent global consensus statements have revised the conceptual framework of sarcopenia, placing greater emphasis on early case finding and repeated assessment in high-risk populations. The updated AWGS 2025 consensus emphasizes early case finding and repeated assessment of sarcopenia in high-risk older adults, with HGS recommended as the first-line screening tool [14]. Even in the absence of overt muscle weakness, individuals with relevant medical risk factors are considered at risk for sarcopenia and are recommended to undergo periodic reassessment and timely muscle health interventions. Hip fracture is a prototypical geriatric syndrome in this context, functioning both as a consequence of and a precipitating factor for sarcopenia, placing patients in a highly vulnerable state characterized by acute stress, immobilization, inflammation, and nutritional decline. Accordingly, older adults with hip fractures are recognized as a priority population for early evaluation and proactive intervention.

Against this background, the observed pattern of greater estimated effects of FIRM among patients with low HGS and, to a lesser extent, among those with sarcopenia, across the unadjusted and adjusted models, highlights muscle strength as a clinically relevant and potentially actionable marker for prioritizing intensive rehabilitation after hip fracture. Within the AWGS 2025 framework, low HGS without concurrent muscle mass assessment is classified as possible sarcopenia and may be interpreted as functional sarcopenia to prompt early identification and intervention [14]. Notably, even patients identified solely by low HGS appeared to derive greater benefits from FIRM than from conventional rehabilitation. Given the limited feasibility of routine muscle mass assessment in postoperative settings [26, 38], these findings suggest the potential utility of HGS as a simple bedside screening tool to guide stratified rehabilitation, whereby patients with impaired muscle strength may be prioritized for integrated programs such as FIRM to optimize functional recovery.

In the fully adjusted models, cognitive impairment, comorbidity burden, and pre-fracture functional status were independently associated with recovery. These findings are consistent with prior evidence indicating that these factors are important determinants of functional recovery after hip fracture [10, 11]. Although cognitive impairment and comorbidity burden are largely non-modifiable, several related factors, including medication optimization, delirium prevention and management, early mobilization, and individualized goal setting, are potentially modifiable and may influence rehabilitation responsiveness [10]. Structured multidisciplinary rehabilitation has been shown to improve mobility and ADL outcomes even in patients with cognitive impairment, particularly when interventions are tailored to individual cognitive and physical capacities [33]. Appropriate management of comorbid conditions may also facilitate participation in rehabilitation and contribute to improved recovery trajectories; thus, these conditions should not be considered barriers to meaningful rehabilitation gains. These findings underscore the importance of comprehensive geriatric assessment and individualized, multidisciplinary rehabilitation strategies that account for both baseline vulnerability and modifiable clinical factors in optimizing functional recovery after hip fractures.

This study has several limitations. First, this secondary analysis was not powered for sarcopenia-stratified comparisons, which limited the statistical power of formal interaction testing. Accordingly, the treatment-by-sarcopenia interaction terms did not consistently reach statistical significance despite internally consistent stratum-specific patterns, and these findings should therefore be interpreted as exploratory and hypothesis-generating. Second, muscle mass measurements were unavailable for a substantial proportion of participants, potentially leading to misclassification of sarcopenia status. Participants with low HGS but missing muscle mass data were classified as having possible sarcopenia; such non-differential misclassification would likely bias the results toward the null, potentially underestimating true differences across sarcopenia severity. Therefore, the observed effect estimates may represent conservative estimates of the true associations. Third, the independent or additive effects of structured nutritional interventions could not be evaluated. Fourth, attrition during the 12-month follow-up period was substantial, which may have introduced bias if loss to follow-up was associated with prognosis. Additionally, recruitment from tertiary rehabilitation centers may limit generalizability.

Despite these limitations, our findings suggest that integrated multidisciplinary rehabilitation may provide greater functional benefits than conventional rehabilitation in older adults with hip fractures, particularly those with low muscle strength. Low muscle strength may not indicate limited rehabilitation potential but may help identify a vulnerable subgroup that may derive greater benefit from intensive, integrated rehabilitation. Future prospective studies that stratify patients according to sarcopenia status and compare conventional and enhanced integrated rehabilitation protocols may help to further refine the optimal strategies for this high-risk population.

## Conclusions

In this secondary analysis of a multicenter RCT, older adults with hip fractures and low HGS may derive greater functional benefits from FIRM than from conventional rehabilitation, whereas the corresponding effects among participants with sarcopenia were less consistent and attenuated in the fully adjusted models. Impaired muscle strength may help identify patients who are likely to benefit from intensive multidisciplinary interventions. These findings suggest the potential value of a targeted and stratified rehabilitation strategy in which baseline muscle strength is used to proactively identify patients most likely to benefit from integrated rehabilitation programs. Early screening for low HGS and prioritization of such patients for intensive integrated rehabilitation may help improve functional recovery after hip fractures.

## Supplementary Information

Below is the link to the electronic supplementary material.

Supplementary file1 (DOCX 568 KB)

## Data Availability

Data are available on reasonable request.
