# Supplementary material for: Integrated rehabilitation and ambulatory recovery in older adults with hip fracture according to muscle strength and sarcopenia status: a secondary analysis of a randomized controlled trial
Source: Eur Geriatr Med. 2026 Aug 20;17(5):2823–33. doi: 10.1007/s41999-026-01587-8 (PMC13755033; doi:10.1007/s41999-026-01587-8)
Supplement: Supplementary file 1 [file 41999_2026_1587_MOESM1_ESM.docx]

| **Table S1. Characteristics by sarcopenia categories among participants with available HGS data.** | | | | |
| --- | --- | --- | --- | --- |
| **Variable** | **Non-sarcopenia (n = 48)** | **Possible sarcopenia (n = 83)** | **Sarcopenia (n = 63)** | ***P-*value^a^** |
| Age, mean (SD), years | 78.8 ± 5.6 | 81.7 ± 7.0 | 82.6 ± 6.9 | 0.009^c^ |
| Sex, n (%) (female) | 37 (77.1) | 64 (77.1) | 46 (73.0) | 0.824 |
| Height, mean (SD), cm | 159.5 ± 6.6 | 154.9 ± 8.2 | 154.3 ± 9.5 | <0.01^b,c^ |
| Weight, mean (SD), kg | 61.4 ± 7.9 | 54.6 ± 9.2 | 48.9 ± 8.3 | <0.001^b,c,d^ |
| BMI, mean (SD), kg/m^2^ | 24.2 ± 3.3 | 22.7 ± 3.2 | 20.6 ± 3.5 | <0.05^b,c,d^ |
| ASMI, mean (SD), kg/m^2^ (n = 119) |  |  |  |  |
| Male | 7.2 ± 1.0 | 7.5 ± 0.4 | 5.4 ± 0.7 | <0.001^c,d^ |
| Female | 6.4 ± 0.8 | 6.1 ± 0.2 | 4.8 ± 0.7 | <0.001^c,d^ |
| HGS, mean (SD), kg (n = 194) |  |  |  |  |
| Male | 31.6 ± 4.0 | 18.4 ± 6.0 | 19.0 ± 4.2 | <0.001^b,c^ |
| Female | 21.7 ± 3.6 | 12.4 ± 3.4 | 11.7 ± 3.2 | <0.001^b.c^ |
| Rehabilitation group, n (%) (FIRM) | 26 (54.2) | 39 (47.0) | 39 (61.9) | 0.302 |
| Fracture type, n (%) |  |  |  | 0.273 |
| Femoral neck | 21 (43.8) | 27 (32.5) | 31 (49.2) |  |
| Intertrochanteric | 24 (50.0) | 52 (62.7) | 28 (44.4) |  |
| Subtrochanteric | 3 (6.2) | 4 (4.8) | 4 (6.3) |  |
| Operation type, n (%) |  |  |  | 0.034 |
| Hemiarthroplasty | 18 (37.5) | 48 (57.8) | 24 (38.1) |  |
| Total hip arthroplasty | 3 (6.2) | 2 (2.4) | 7 (11.1) |  |
| Internal fixation | 27 (56.2) | 33 (39.8) | 32 (50.8) |  |
| Pre-fracture Koval, mean (SD), score | 1.3 (0.7) | 1.7 (1.0) | 2.0 (1.6) | 0.008^c^ |
| Pre-fracture FAC, mean (SD), score | 4.8 (0.6) | 4.7 (0.6) | 4.5 (0.9) | 0.44^c^ |
| Pre-fracture gait impairment, n (%) | 2 (4.2) | 5 (6.0) | 11 (17.5) | 0.023 |
| Previous history of hip fracture, n (%) | 1 (2.1) | 1 (1.2) | 9 (14.3) | 0.002 |
| Cognitive impairment, n (%) | 10 (20.8) | 35 (42.2) | 35 (55.6) | 0.001 |
| Osteoporosis, n (%) | 26 (54.2) | 63 (75.9) | 49 (77.8) | 0.011 |
| Charlson Comorbidity Index, n (%) |  |  |  | 0.001 |
| None | 14 (29.2) | 39 (47.0) | 15 (23.8) |  |
| Mild (1–2 conditions) | 22 (45.8) | 37 (44.6) | 29 (46.0) |  |
| Moderate (3–4 conditions) | 3 (6.3) | 5 (6.0) | 10 (15.9) |  |
| Severe (≥ 5 conditions) | 9 (18.8) | 2 (2.4) | 9 (14.3) |  |
| Postoperative complications, n (%) | 3 (6.2) | 1 (1.2) | 7 (11.1) | 0.037 |
| Length of hospital stay, mean (SD), days | 22.3 ± 5.8 | 23.6 ± 7.0 | 25.1 ± 5.9 | 0.067^c^ |
| Days to surgery, mean (SD) | 2.4 ± 2.5 | 3.1 ± 3.1 | 3.4 ± 3.3 | 0.274^c^ |
| Days to rehabilitation, mean (SD) | 7.5 ± 3.1 | 7.5 ± 2.8 | 8.6 ± 4.2 | 0.134^d^ |
| Discharge destination, n (%) |  |  |  | 0.003 |
| Home | 35 (72.9) | 36 (43.4) | 30 (47.6) |  |
| Institution | 13 (27.1) | 47 (56.6) | 33 (52.4) |  |
| Independent ambulation at 12 months, n (%) (n = 116) | 31 (86.1) | 20 (64.5) | 28 (57.1) | 0.016 |
| Participants without muscle mass measurements were not classified as having sarcopenia, even if they had low handgrip strength. Sarcopenia status could not be determined in 9 participants due to missing baseline handgrip strength and muscle mass measurements.  HGS, handgrip strength; BMI, body mass index; ASMI, appendicular skeletal muscle mass index; Fragility Fracture Integrated Rehabilitation Management; FAC, functional ambulation category.  ^a^ P-values were calculated using one-way analysis of variance or the χ² test, as appropriate.  ^b^ Non-sarcopenia vs. possible sarcopenia.  ^c^ Non-sarcopenia vs. sarcopenia.  ^d^ Possible-sarcopenia vs. sarcopenia. | | | | |

**Figure S2.** Age- and sex- adjusted effect of FIRM on 12-month independent ambulation. Panel A presents Model 1 (sarcopenia vs non-sarcopenia), Panel B presents Model 2 (three-level AWGS 2025 sarcopenia classification), and Panel C presents Model 3 (low vs normal handgrip strength). Treatment effects of FIRM compared with conventional rehabilitation are shown as stratum-specific odds ratios (ORs), risk differences (RDs), and numbers needed to treat (NNTs). FIRM, Fragility Fracture Integrated Rehabilitation Management.

| **Table S3. Baseline characteristics of participants with and without 12-month follow-up.** | | | |
| --- | --- | --- | --- |
| **Variable** | **Follow-up group**  **(n = 108)** | **No follow-up group**  **(n = 95)** | ***P-*value** |
| Age, mean (SD), years | 80.6 (6.7) | 82.5 (6.6) | 0.047 |
| Sex, n (%) (female) | 93 (78.2) | 62 (73.8) | 0.583 |
| Height, mean (SD), cm | 155.6 (8.0) | 155.9 (9.1) | 0.776 |
| Weight, mean (SD), kg | 55.4 (9.7) | 52.4 (9.6) | 0.028 |
| BMI, mean (SD), kg/m^2^ | 22.9 (3.4) | 21.6 (3.7) | 0.011 |
| ASMI, mean (SD), kg/m^2^ (n = 119) |  |  |  |
| Male | 6.3 (1.3) | 6.2 (1.2) | 0.878 |
| Female | 5.5 (1.0) | 5.2 (1.1) | 0.209 |
| HGS, mean (SD), kg (n = 194) |  |  |  |
| Male | 24.0 (6.8) | 19.1 (7.3) | 0.020 |
| Female | 15.1 (5.6) | 13.7 (5.0) | 0.130 |
| Rehabilitation group, n (%) (FIRM) |  |  |  |
| Fracture type, n (%) |  |  | 0.099 |
| Femoral neck | 55 (46.2) | 27 (32.1) |  |
| Intertrochanteric | 57 (47.9) | 53 (63.1) |  |
| Subtrochanteric | 7 (5.9) | 4 (4.8) |  |
| Operation type, n (%) |  |  | <0.001 |
| Bipolar hemiarthroplasty | 41 (34.5) | 58 (69.0) |  |
| Total hip arthroplasty | 11 (9.2) | 1 (1.2) |  |
| Internal fixation | 67 (56.3) | 25 (29.8) |  |
| Pre-fracture Koval, mean (SD), score | 1.8 (1.33) | 1.6 (0.9) | 0.194 |
| Pre-fracture FAC, mean (SD), score | 4.6 (0.7) | 4.7 (0.7) | 0.354 |
| Pre-fracture gait impairment, n (%) | 13 (10.9) | 7 (8.3) | 0.711 |
| Previous history of hip fracture, n (%) | 10 (8.4) | 2 (2.4) | 0.136 |
| Cognitive impairment, n (%) | 67 (56.3) | 50 (59.5) | 0.754 |
| Osteoporosis, n (%) | 82 (68.9) | 65 (77.4) | 0.242 |
| Charlson Comorbidity Index, n (%) |  |  | 0.845 |
| None | 39 (32.8) | 31 (36.9) |  |
| Mild (1–2) | 55 (46.2) | 37 (44.0) |  |
| Moderate (3–4) | 12 (10.1) | 6 (7.1) |  |
| Severe (≥ 5) | 13 (10.9) | 10 (11.9) |  |
| Postoperative complication, n (%) | 9 (7.6) | 2 (2.4) | 0.197 |
| Length of hospital stay, mean (SD), days | 23.9 (6.0) | 23.4 (6.8) | 0.556 |
| Days to surgery, mean (SD), | 2.9 (2.6) | 3.2 (3.5) | 0.517 |
| Days to rehabilitation, mean (SD), | 8.3 (3.8) | 7.1 (2.3) | 0.010 |
| Discharge location, n (%) |  |  | 0.001 |
| Home | 74 (62.2) | 31 (36.9) |  |
| Institution | 45 (37.8) | 53 (63.1) |  |
| BMI, body mass index; ASMI, appendicular skeletal muscle mass index; HGS, handgrip strength; FIRM, Fragility Fracture Integrated Rehabilitation Management.; FAC, functional ambulation category. | | | |

**Conventional rehabilitation**

**Favors FIRM**

**A**

**C**

**Conventional rehabilitation**

**Favors FIRM**

**B**

**Conventional rehabilitation**

**Favors FIRM**

**Figure S4.** Sensitivity analyses of the effect of FIRM on 12-month independent ambulation among participants with available baseline HGS data under worst-case assumptions. In the worst-case scenario, all participants lost to follow-up were assumed to be non-ambulatory. Panel A shows unadjusted estimates; Panel B shows age- and sex-adjusted estimates; and Panel C shows fully adjusted estimates. Treatment effects of FIRM compared with conventional rehabilitation are presented as stratum-specific odds ratios (ORs), risk differences (RDs), and numbers needed to treat (NNTs). FIRM, Fragility Fracture Integrated Rehabilitation Management.

**A**

**Conventional rehabilitation**

**Favors FIRM**

**Conventional rehabilitation**

**Favors FIRM**

**B**

**C**

**Conventional rehabilitation**

**Favors FIRM**

**Figure S5.** Sensitivity analyses of the effect of FIRM on 12-month independent ambulation among participants with available baseline HGS data under best-case assumptions. In the best-case scenario, all participants lost to follow-up were assumed to be ambulatory. Panel A shows unadjusted estimates; Panel B shows age- and sex-adjusted estimates; and Panel C shows fully adjusted estimates. Treatment effects of FIRM compared with conventional rehabilitation are presented as stratum-specific odds ratios (ORs), risk differences (RDs), and numbers needed to treat (NNTs). FIRM, Fragility Fracture Integrated Rehabilitation Management.
